# A real-world preventive primary care model for cardiorenal metabolic disease: clinical impact of a personalised care approach in Harrow, North West London

**DOI:** 10.1186/s12882-026-05040-7

**Published:** 2026-05-29

**Authors:** R. Dattani, R. Rahman, S. Siddik, V. Brookman, A. Kelshiker, J. Shah, A. H. Frankel, M. Joshi, K. Johal

**Affiliations:** 1 Harrow Network Partners Ltd, London, UK; 2 Imperial College London, London, UK; 3 Imperial College NHS Trust, London, UK; 4 Imperial College Health Partners, London, UK; 5 Sphere PCN, London, UK; 6 Healthsense PCN, London, UK; 7 Harrow Collaborative PCN, London, UK; 8 London North West NHS Trust, London, UK

**Keywords:** Personalised healthcare, Cardiorenal–metabolic disease, Chronic kidney disease prevention, Primary care pathway, Patient empowerment

## Abstract

**Background:**

Cardiorenal metabolic (CRM) disease, is identified by the co-location of multiple disorders including obesity, diabetes, hypertension, cardiovascular disease and chronic kidney disease (CKD). Early intervention is essential to slow CKD progression, reduce cardiovascular risk, and improve quality of life. The Harrow CRM Hub project established a personalised, multidisciplinary pathway to identify high-risk patients, optimise clinical management, and provide access to lifestyle and psychosocial support. This paper reports on the clinical outcomes achieved within the first year of implementation.

**Methods:**

A comprehensive logic model was co-developed to guide the design, delivery, and evaluation of the Harrow CRM programme. Two EHR-identified cohorts were invited: (1) adults aged 20–80 years with BMI >27.5–30 kg/m² (ethnicity-dependent) and non-diabetic hyperglycaemia ± hypertension (CRM Stage 2); and (2) adults with diabetes ± CKD or CVD (CRM Stage 4). Pre visit health questionnaire – using digital tools enabled detailed pre visit updates and tests. Protected consultations (lasting 30 to 45 minutes) followed a structured EHR template incorporating guideline-based optimisation of pharmacotherapy, risk calculators, and co-created lifestyle care plans. Data were extracted for paired analysis of systolic BP, HbA1c, and weight. A qualitative evaluation was undertaken to explore patient and staff experiences of the CRM pathway.

**Results:**

Thus far, between November 2024 and September 2025, 2,641 patients were reviewed, with 2,300 included in paired analysis. Across the full cohort, mean changes were −3.65 mmHg in systolic BP (median −2.0 mmHg), −1.03 mmol/mol in HbA1c (median 0.0 mmol/mol), and −0.46 kg in weight (median 0.0 kg) (all p<0.001). For those with an improvement only - an average improvement of −14.12 mmHg (n=1,279) and an average deterioration of +10.61 mmHg among those whose readings worsened (n=895). HbA1c values showed a mean cohort wide reduction of −1.03 mmol/mol (median 0.0 mmol/mol), with mean changes of −8.08 mmol/mol among improvers (n=785) and +5.22 mmol/mol among those with deterioration (n=762). Weight trends showed a mean overall reduction of −0.46 kg (median 0.0 kg), comprising an average improvement of −3.63 kg among improvers (n=1,124) and deterioration of +3.93 kg among those with deterioration (n=761). Among those with paired readings, 33.4% achieved ≥5% BP reduction and 19.7% achieved ≥10%; 19.8% achieved ≥5% HbA1c improvement and 12.7% ≥10%; and 9.6% achieved ≥5% weight loss and 2.7% ≥10%. Overall, 73.9% improved in ≥1 parameter, while 10.4% improved across BP, HbA1c, and weight simultaneously. This real world review identified patients with improvements and deterioration in their health parameters. Qualitative findings showed patients valued extended consultations and holistic discussions, with vast majority of patients reporting greater understanding of their health and feeling more confident to manage it. A staff survey (n=14) provided supportive but preliminary quantitative evidence of having greater confidence in delivering CRM clinics and increased ability to access multidisciplinary expertise.

**Conclusion:**

A personalised, multidisciplinary CRM model embedded within primary care was associated with statistically and clinically significant improvements in blood pressure, glycaemic control, and weight in a large, ethnically diverse population. Patients and clinicians both reported greater engagement, confidence, and satisfaction. The approach combining structured identification, extended consultations, co-produced care plans, and workforce education demonstrates a scalable, sustainable pathway to slow CKD progression, reduce CVD risk, and enhance patient wellbeing across diverse communities.

**Supplementary Information:**

The online version contains supplementary material available at 10.1186/s12882-026-05040-7.

## Introduction

Cardiorenal metabolic (CRM) disease describes the convergence of multiple health conditions including obesity, type 2 diabetes, hypertension, cardiovascular disease (CVD), and chronic kidney disease (CKD). The clustering of these conditions reflects shared pathophysiological mechanisms, including insulin resistance, endothelial dysfunction, inflammation, and maladaptive neurohormonal activation [1]. Together, they contribute disproportionately to premature mortality and morbidity worldwide,. The global prevalence of CKD is estimated at nearly 10% of the population, with cardiovascular complications remaining the leading cause of death in this group [2]. In the UK, CKD affects approximately one in ten adults, but recognition rates remain suboptimal, with many patients only identified at later stages of disease when opportunities for prevention and halting CKD progression and CVD development have been missed [3]. The increasing incidence of diabetes, hypertension and obesity, particularly in younger and ethnically diverse populations, underscores the urgency of addressing CRM disease as a single, integrated challenge [4]. Recognising the interconnectivity and shared mechanisms between these conditions is essential not only to raise awareness among multidisciplinary clinical teams but, more critically, to empower patients with an understanding of how one condition can influence the development, progression, or even potential reversibility of others.

**Fig. 1:**
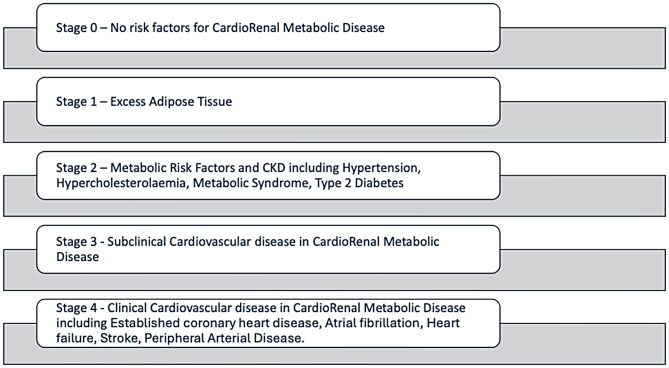
Conceptual framework illustrating progressive stages of Cardiorenal Metabolic (CRM) disease, from absence of risk factors to established cardiovascular disease. Adapted conceptually from Vaduganathan M et al., Circulation 2023;148:907–924. 24

The cardiorenal–metabolic continuum highlights how metabolic and vascular dysfunction develop progressively, often long before clinical disease becomes apparent. Early stages are driven by excess adiposity, impaired glucose tolerance, and hypertension, which collectively increase the risk of both CKD and cardiovascular disease. As risk factors accumulate, patients transition from subclinical to overt cardiovascular and renal disease. (Fig. 1) Each condition in CRM syndrome independently reduces survival. Grade 3 obesity (BMI 40–45 kg/m2) is linked to a reduction in median life expectancy of 8–10 years [5]. Individuals with diabetes live on average 13 years less than non-diabetic counterparts, while patients with stage 4 CKD (GFR 15-29 ml/min/1.73m2) may have > 20-year-shorter life expectancy compared with those with normal or mildly impaired kidney function [6, 7]. It is therefore not surprising that when combined, individuals with CRM have much higher morbidity and mortality rates, placing significant strain on health services. The financial burden is considerable, with annual NHS costs estimated at £12 billion for CVD, £14 billion for diabetes, and £7 billion for CKD [8–10].

The CRM public health crisis is exacerbated by the poor recognition of the link between CRM diseases in clinical practice. People living with these diseases often require expertise from multiple specialists, including cardiologists, nephrologists, endocrinologists, hepatologists and primary care physicians. However, multidisciplinary collaboration remains inconsistent, and care is frequently delivered through single-specialty services. Whilst current international guidelines provide clear evidence-based frameworks for managing these conditions, they are frequently delivered in silo. The Kidney Disease: Improving Global Outcomes (KDIGO) 2022 guidelines emphasise the importance of early detection, risk stratification, and the use of Reno protective therapies including SGLT2 inhibitors and renin–angiotensin–aldosterone system blockade [11]. Similarly, the American Diabetes Association (ADA) and European Society of Cardiology (ESC) guidelines highlight integrated management of diabetes, cardiovascular disease, and renal impairment as a cornerstone of reducing long-term complications [12, 13]. NICE guidance within the UK also prioritises optimisation of blood pressure, glycaemic control, and lipid management, while encouraging patient education and self-management support. However, despite these clear recommendations, implementation in routine practice is often fragmented, with disease-specific pathways delivered in silos and constrained by short consultation times. This fragmented approach results in duplicated appointments, conflicting advice, and unnecessary patient burden, resulting in limited attention to multimorbidity, lifestyle behaviours, and the psychosocial determinants of health [14–17]. Patients frequently report receiving conflicting advice from different clinicians, inconsistent access to lifestyle and psychosocial support, and inadequate emphasis on prevention. For clinicians, short consultation times and competing priorities, alongside limited prior experience in health coaching and personalised care techniques further limit the ability to provide holistic care. These gaps are associated with poorer clinical outcomes, reduced patient satisfaction, and higher long-term healthcare utilisation [18]. There is therefore an urgent need to develop a unified, integrated model of care to improve clinical efficiency and provide patients with understandable, person centred management across the CRM spectrum, with an ultimate goal to stop the development and progression of CKD, reduce cardiovascular morbidity and deaths and improve quality of life [19].

The London borough of Harrow serves a socially diverse and ethnically diverse population n of around 261,000 and faces a significant burden of poor CRM health,. There is a higher prevalence of obesity (12%) and diabetes (8.4%) in this borough, compared to the national prevalence of 11.2%% and 6.4% respectively, highlighting the need to provide patients with an integrated model of care [20]. 

Personalised healthcare has emerged as a promising strategy to overcome the fragmented care currently offered to individuals with CRM disease. Defined broadly, it involves tailoring interventions to the unique risk factors, preferences, and circumstances of each individual, while also addressing broader determinants of health. Within primary care in Harrow, a personalised approach has included proactive case finding, risk stratification tools, extended consultations, and collaborative care planning with the patient that encompasses pharmacological (medicines) optimisation, lifestyle modification, and psychosocial support [21]. Evidence suggests that patients feeling informed, motivated, and confident enough to manage their own health is strongly associated with improved outcomes across long-term conditions, including CKD with digital technology identified to be capable of empowering patients to self monitor their individual progress through identified parameters. Embedding personalised care within multidisciplinary teams may also enhance professional fulfilment and sustainability by supporting new skills in health coaching, lifestyle medicine, and collaborative working [22]. 

Policy frameworks in the UK, such as the NHS Long Term Plan and the Core20PLUS5 approach to tackling health inequalities, explicitly prioritise personalised and preventative care for long-term conditions [23, 24]. However, translating these ambitions into practical, scalable models remains a challenge. Few studies have integrated the prevention of CKD progression into a broader CRM care pathway, and even fewer have combined quantitative evaluation of outcomes with qualitative insights from patients, clinicians, and operational staff. There is also a recognised need for workforce development and training tools to equip primary care teams with the knowledge and confidence to deliver personalised care consistently.

Supported by a grant from NHSE (London) as part of the Renal 3Ps project, the Harrow CRM project aims to empower patients through education, personalised care, and multidisciplinary collaboration. Established across five primary care networks in Harrow, North West London it sought to implement a structured, personalised, multidisciplinary pathway for patients at risk of, or living with, CRM disease from inception in November 2024 to end of December 2025 (ongoing at the time of writing). The model combined systematic risk stratification, extended consultations with prescribing clinicians, and co-created personalised care plans. It incorporated proactive referral and signposting to lifestyle, psychosocial, and voluntary sector support, thereby extending into local community services, alongside ongoing follow-up and monitoring. This evaluation provides an account of the Harrow CRM Hub and showcases a model of intervention delivered and embedded into routine primary care for the benefit of patients with early CRM. The findings offer important insights into both the potential impact and the practical challenges of such an approach, with relevance for future scale-up of CRM pathways and prevention of CKD progression.

## Methods

**Fig. 2:**
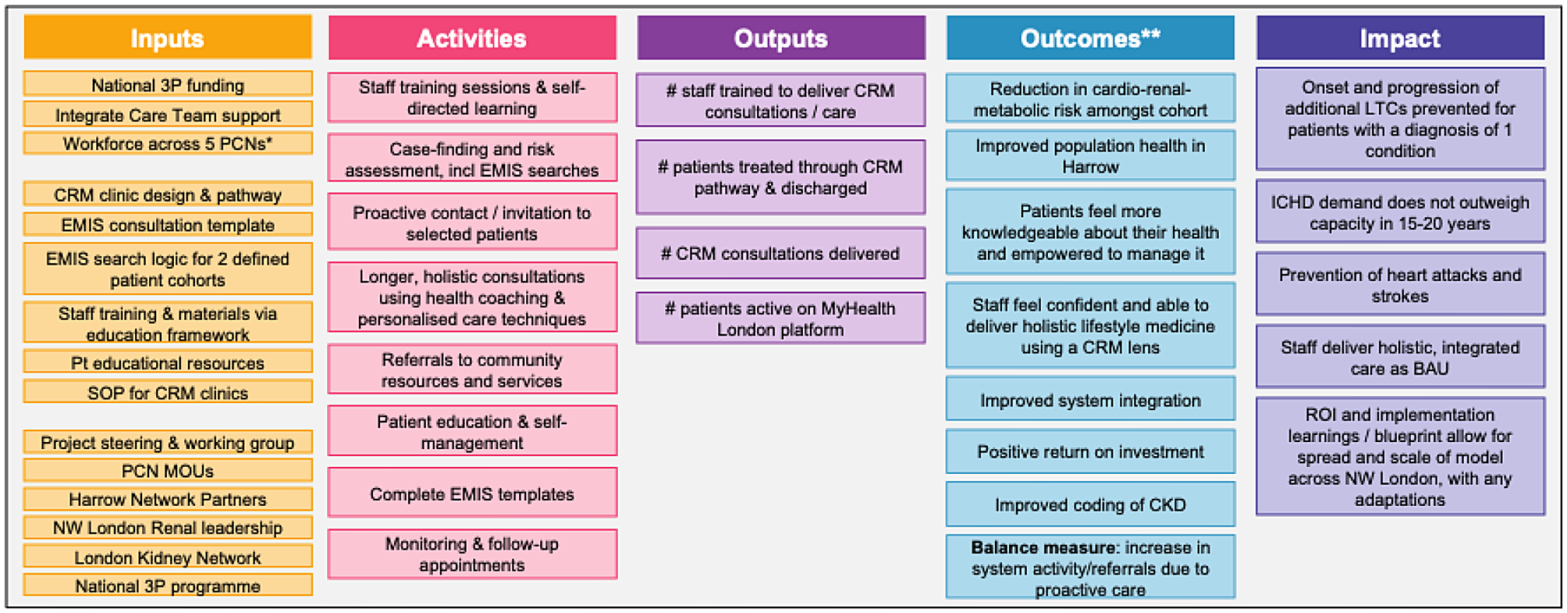
Logic model for the Harrow Cardiorenal–Metabolic (CRM) programme. The model summarises identified/ intended key inputs, activities, outputs, outcomes, and impacts of the CRM pathway

A comprehensive logic model was developed collaboratively at project inception to guide the design, delivery, and evaluation of the Harrow CRM programme (Fig. 2). The model was co-produced through a series of multi-stakeholder workshops in 2024 and refined via one-to-one interviews with members of the CRM Steering Group. It outlined the key inputs (such as national 3P funding, integrated care support, EHRs templates, and workforce development across five primary care networks), the planned activities (including staff training, case finding, extended consultations, and personalised care planning), and the expected outputs (CRM consultations delivered, staff trained, and patients engaged through digital platforms). These elements were aligned to measurable outcomes, notably improved health metrics, enhanced staff confidence, greater patient activation, the longer term impact of reducing CRM risk, slowing CKD progression, and embedding an integrated model of care across North West London. The logic model provided the operational and evaluative framework for the CRM pathway, ensuring consistency across participating practices and enabling monitoring of both clinical and implementation outcomes. As a result, a detailed educational framework was developed to address gaps in existing training resources and to ensure shared ownership of the CRM approach. This framework defined how and why the Harrow model would differ from conventional care, while providing the key enabling tools required to support implementation at the frontline. It represented a co-designed, dynamic, and evolving approach that embedded learning, feedback, and continual improvement into the delivery process.

CRM staging was based on predefined criteria incorporating metabolic risk, glycaemic status, and the presence of cardiovascular or renal disease, aligned with published cardiorenal metabolic frameworks. By use of the Egton Medical Information Systems (EMIS) – two key cohorts of patients were identified:

1. Cohort 1 – Age 20–80, BMI > 27.5–30 (dependent on ethnicity), Non diabetic hyperglycaemia +/- Hypertension – thereby identifying patients in Stage 2 CRM (Fig. 1).
2. Cohort 2 – Age 20–80, DM with CVD and +/- known CKD (1–5) – thereby identifying patients in Stage 4 CRM (Fig. 1).

Patients who were housebound, with terminal illness, advanced disease or already receiving specialist management were excluded.

Implemented sequentially across five primary care networks (PCNs) in Harrow—Harrow Collaborative, Harrow East, Healthsense, Health Alliance, and Sphere, eligible patients were proactively invited to a dedicated CRM appointment. Prior to the CRM appointment invited individuals received a pre-clinic health questionnaire via AccuRx (a digital healthcare communication tool used in the NHS), and were signposted to digital platforms including My Health London, “Calculate your heart age – NHS”, NWL Lifestyle leaflet and Metabolic Syndrome page on heartuk.org.uk. The aim was to encourage patients to become empowered/motivated to attend by utilising pre-existing publicly available tools.

To ensure accuracy and efficiency in data capture, the pre-clinic questionnaire was directly linked to Quality and Outcomes Framework (QOF)–related SNOMED codes, reducing duplication of effort and aligning with contractual practice requirements. Patients were able to enter their own blood pressure, waist circumference, height, and weight, as well as provide information on ethnicity, employment status, smoking and alcohol use, and level of physical activity. For those who were digitally able, this was an important stage of patient activation and engagement ahead of the forthcoming lifestyle consultations when these metrics were explored in more detail and incorporated into their lifestyle care plan. These responses automatically populated a detailed CRM EHR template, co-designed by the project team to cover CRM-related conditions, relevant investigations (blood and urine tests), and embedded clinical tools such as QRISK2/3, Kidney Failure Risk Equation (KFRE), and Fib-4, alongside prompts addressing the six pillars of lifestyle medicine. The six pillars of lifestyle medicine include nutrition, physical activity, sleep, stress management, avoidance of harmful substances, and social connection. These domains provide a structured framework for addressing modifiable behavioural risk factors and underpin the development of personalised care plans within the CRM pathway. Early in implementation, an underutilised SNOMED code “Metabolic syndrome” was adopted to systematically identify patients enrolled in the Cardiorenal–Metabolic programme. For individuals who did not complete the electronic questionnaire, the same information was collected manually by navigators in advance of their clinical appointment.

First appointment consultations were delivered by prescribing clinicians, typically lasting up to 30–45 min and one follow-up appointment of 10–15 min duration (3–6 months later). Key elements included recognising the importance of “personalising” information, demonstrating and reviewing trends over time graphically. This engaged patients in drawing upon their personal experiences and knowledge of how better to manage their health including addressing non adherence to medication. Key elements included during the first consultation included:

- Review of recent investigations (blood pressure, HbA1c, lipid profile, kidney function, urinary albumin-to-creatinine ratio, body mass index, waist circumference). Trends over time – graphical charts.
- Use of validated risk calculators (QRISK2/3, Heart Age, Kidney Failure Risk Equation, and Fib-4 where relevant).
- Optimisation of pharmacological therapy in line with NICE and KDIGO guidelines, including initiation of SGLT2 inhibitors, GLP-1 receptor agonists, renin–angiotensin–aldosterone system blockade, and statins where indicated.
- Co-creation of a personalised lifestyle plan, covering diet, physical activity, sleep, mental health, and social wellbeing.
- Referral or signposting to weight management services, physiotherapy, psychological therapies, welfare advice, and voluntary sector or digital resources, where appropriate.

Primary care clinicians could directly access remote advice from nephrology, cardiology or endocrinology specialists, involved in the development of the CRM pathway, as needed.

Follow-up assessments were conducted at approximately 3–6 months after the initial consultation, with repeat measurements used for paired analysis. Follow-up included care coordinator check-in calls, repeat measurements at three- to six-month intervals, with a follow up appointment in the CRM clinic. Clinical outcomes were extracted from electronic health records, with paired baseline and follow-up data analysed for changes in blood pressure, HbA1c, weight, and waist circumference in the first instance.

### Patient and staff insights

To explore experiences of the pathway, we conducted semi-structured interviews with 18 patients and 22 staff members (GPs, pharmacists, physician associates, nurses, care coordinators, and receptionists). In addition, 14 staff completed an anonymous online survey. Both the interviews and online survey were developed for this study. (Supplementary material). Participants were purposively sampled to reflect a range of demographics and professional roles. The questionnaire and interview schedules were developed specifically for this study, informed by existing literature and clinical guidelines. Interviews were conducted in person or virtually, audio-recorded with consent, and transcribed verbatim. Data were analysed thematically using NVivo software, with findings triangulated against survey responses to strengthen validity.

### CRM staff education

In parallel, a multidisciplinary working group of clinicians, managers, and project leads developed a bespoke educational resource to support delivery of CRM care. Existing national and regional resources were mapped against the competencies required for holistic CRM management, and a structured training package was created. Content was organised around three domains:

- Why: epidemiology and population need for integrated CRM care.
- How: operational delivery of the pathway within primary care.
- Me: personalised communication skills and health coaching.

The package included slide sets, case scenarios, EHR templates/searches, and a tiered training framework tailored to healthcare assistants, non-prescribing clinicians, and prescribers. Iterative feedback from pilot sessions informed refinement of the materials.

Monthly review meetings – sharing the data, data completeness and patient review continue - reflecting a truly learning organisation. Collaboration with the public health team also ensured that awareness of CRM was incorporated into MECC (Making Every Contact Count) training for Community Health Champions and allied health professionals (such as social prescribers, health coaches and care co-ordinators) to support health education conversations in the wider community.

Descriptive statistics were used to summarise baseline demographics and data completeness. Analyses were performed using paired data for each parameter, and no imputation of missing data was undertaken. For patients with paired results, paired t-tests were performed to assess changes in continuous outcomes, including blood pressure, HbA1c, weight, and waist circumference. Proportions of patients achieving clinically meaningful thresholds (≥ 5% or ≥ 10% reduction) were calculated. A significance threshold of *p* < 0.05 was applied.

## Results

Between November 2024 and September 2025, 2,641 patients were reviewed within the CRM Hub, of whom 2300 were included in the analysis after exclusion of outliers at the time of writing. Outliers were defined as clinically implausible values or extreme changes between baseline and follow-up that were inconsistent with expected variation, and were excluded following clinical review prior to analysis. The cohort of 2,641 patients was roughly evenly split by gender (51.2% female, 48.8% male) with a mean age of 60.5 years for women and 60.3 years for men.

Baseline completeness was high for blood pressure (97%), HbA1c (96%), and weight (87%), though lower for waist circumference (79%). Data completeness varied across parameters (Table 1).

**Table 1:**
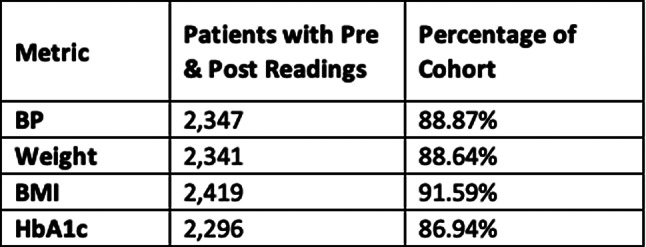
Data completeness for key clinical parameters. Number and percentage of patients with both pre- and post-intervention readings across major clinical metrics

**Fig. 3:**
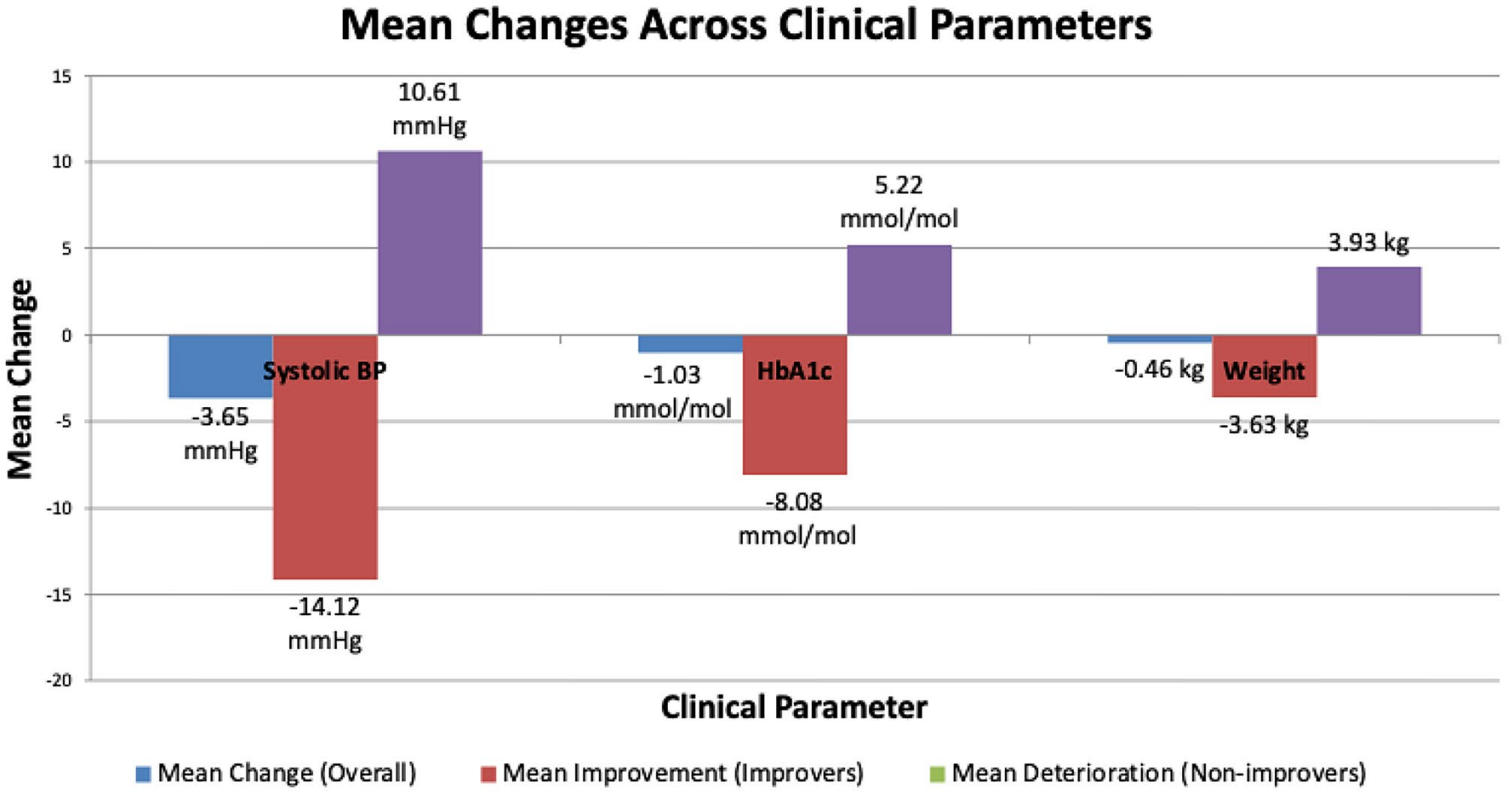
Overall mean change, mean improvement and mean deterioration for systolic blood pressure, HbA1c and weight

Across paired measurements, data completeness ranged from 2,296 to 2,347 patients. Overall, statistically significant improvements were observed across all major parameters (*p* < 0.001). For systolic blood pressure, the mean change across the entire cohort was − 3.65 mmHg (median − 2.0 mmHg). For those with an improvement only - an average improvement of − 14.12 mmHg (*n* = 1,279) and an average deterioration of + 10.61 mmHg among those whose readings worsened (*n* = 895). HbA1c values showed a mean cohort wide reduction of − 1.03 mmol/mol (median 0.0 mmol/mol), with mean changes of − 8.08 mmol/mol among improvers (*n* = 785) and + 5.22 mmol/mol among those with deterioration (*n* = 762). Weight trends showed a mean overall reduction of − 0.46 kg (median 0.0 kg), comprising an average improvement of − 3.63 kg among improvers (*n* = 1,124) and deterioration of + 3.93 kg among those with deterioration (*n* = 761). (Fig. 3)

**Fig. 4:**
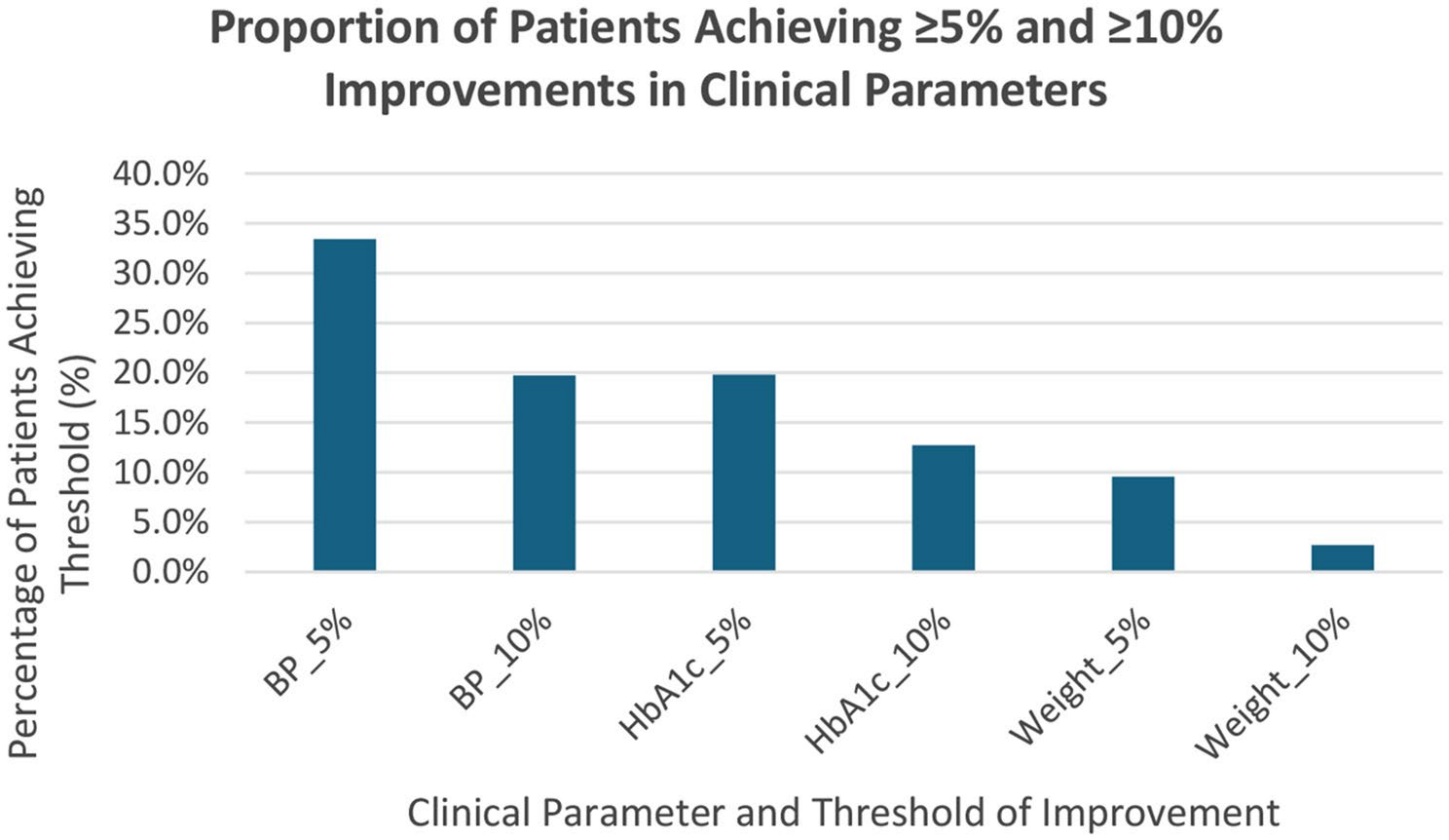
Proportion of patients achieving ≥ 5% and ≥ 10% improvements in clinical parameters: Systolic blood pressure (BP), HbA1c, and weight

Among those with paired systolic blood pressure readings, 33.4% achieved a ≥ 5% reduction and 19.7% achieved a ≥ 10% reduction. For weight, 9.6% achieved a ≥ 5% loss and 2.7% achieved a ≥ 10% loss, while 19.8% of patients with paired HbA1c results achieved a ≥ 5% improvement and 12.7% achieved a ≥ 10% improvement. These findings indicate that around one in three patients achieved meaningful blood-pressure reduction and one in five achieved moderate improvements in glycaemic control within the study period. (Fig. 4)

**Fig. 5:**
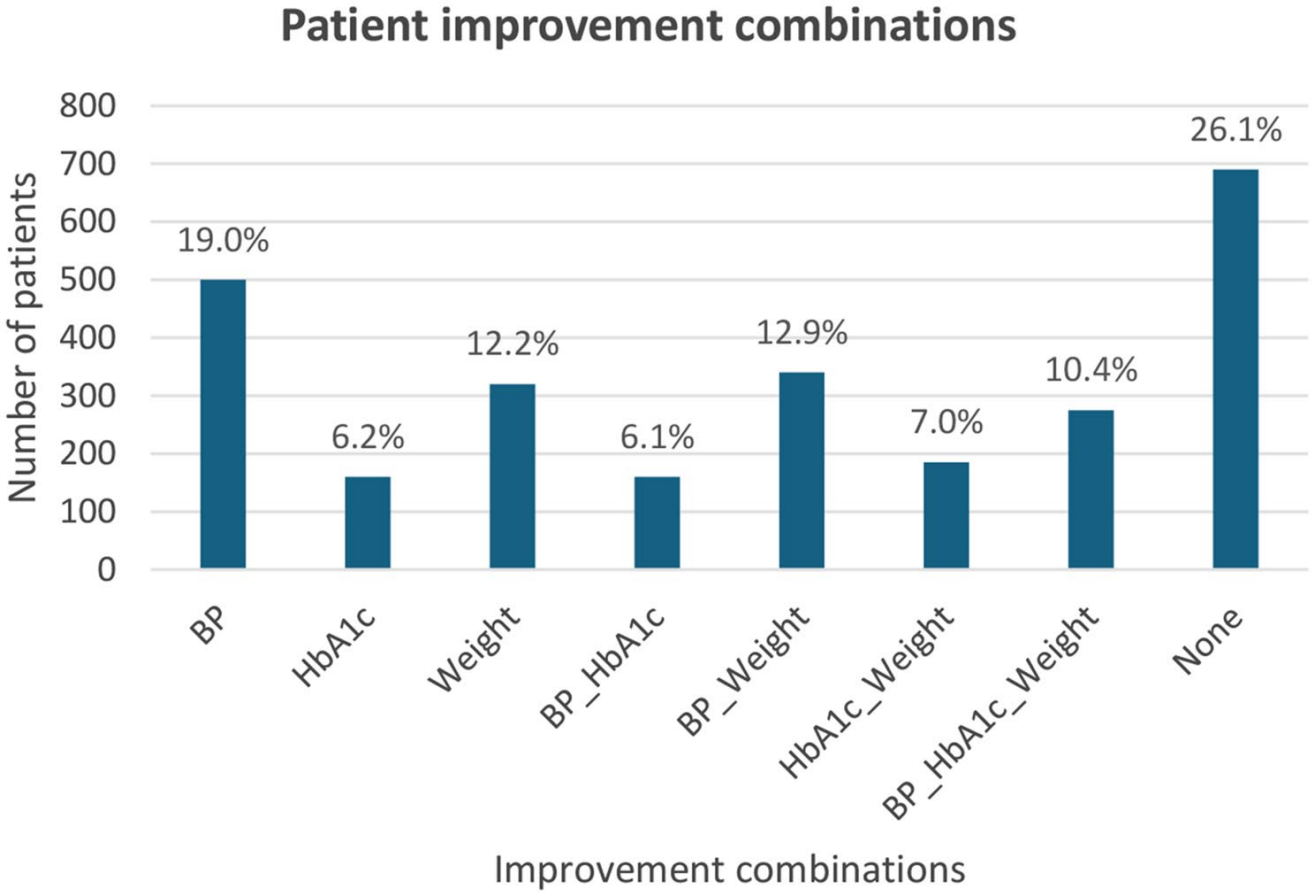
Patient improvement combinations across clinical parameters. Proportion of patients showing improvement in one or more clinical measures

Across combinations of clinical parameters, 19.0% of patients achieved improvement in blood pressure alone, 6.2% in HbA1c alone, and 12.2% in weight alone. Combined improvements were observed in 6.1% for blood pressure and HbA1c, 12.9% for blood pressure and weight, and 7.0% for HbA1c and weight. A total of 10.4% of patients demonstrated improvement across all three parameters (blood pressure, HbA1c, and weight), while 26.1% did not show measurable improvement in any domain. Overall, 73.9% of patients achieved improvement in at least one clinical parameter (Fig. 5).

### Patient and staff insights

A total of 18 patients and 22 staff members participated in semi-structured interviews, with a further 14 staff completing online surveys across the five Harrow Primary Care Networks.

Patients consistently described CRM appointments as a positive and distinct experience compared to usual care, highlighting that longer appointment length helped build trust and enabled more valuable conversations covering lifestyle, mental wellbeing, sleep, and social factors. Key motivators for engagement included a better understanding that lifestyle changes could reverse or reduce future health risks, receiving personalised and culturally tailored advice, and visual explanations of test results that made risk feel tangible and actionable. Many patients valued the sense of accountability created by follow-up appointments and clinician support, and several expressed a desire to avoid or reduce reliance on medication where possible. A recurrent theme was a strong preference for having more knowledge and control over their own health. All but one patient reported increased motivation to make lifestyle changes post-appointment, with patients reporting feeling empowered by understanding their risk and receiving actionable steps. Some patients described the actions they were already taking because of their CRM appointments, showing evidence of behaviour change. Such actions included improved tracking of their own health data and lifestyle changes. Representative comments included: “This is the first time I feel I have medical attention — I no longer feel alone” and “*“They showed an interest in me*,* took time to explain to me*,* to spend that time with me.”*

Staff reported increased confidence in delivering CRM clinics, especially in health coaching and lifestyle conversations, with training identified to help staff navigate sensitive topics like weight, motivation and medication hesitancy. In addition, 71% of staff surveyed (10 of 14) reported increased knowledge in co-morbidity prevention and management. Shadowing and role-playing were cited as the most effective training formats, especially for newer staff. Staff appreciated peer learning and case discussions.

57% of staff survey respondents (8 of 14) reported that their involvement in CRM clinics greatly increased or increased their satisfaction in their role/work. Clinicians repeatedly described the clinics as “more rewarding than routine reviews” and valued observing tangible patient progress over time, with staff reporting a sense of impact and professional satisfaction. 64% of staff survey respondents (9 of 14) felt more able to access the expertise of wider team members outside their practice (e.g. renal consultant). 57% (8 of 14) felt more able to access expertise of wider team members within their practice and gained a greater awareness of local services to signpost or refer patients to.

Despite strong engagement with the CRM model, several operational and practical challenges were identified. Staff noted that patient non-attendance or declines were often linked to limited understanding of the purpose of the CRM appointment or its relevance to their long-term health risks. Competing priorities and limited protected time made it difficult for some clinicians to attend or complete the full CRM training programme. Within consultations, staff reported difficulty in covering the full breadth of clinical review, education, and personalised care planning. Additional challenges included incomplete pre-appointment questionnaires, post-appointment satisfaction surveys, and delays in obtaining repeat blood or urine samples.

Staff expressed uncertainty regarding the long-term continuation of the CRM pathway, citing its resource intensity and the need for sustained funding to maintain delivery at scale. Many emphasised that ongoing patient engagement and measurable clinical improvements would be critical determinants of the model’s sustainability. Several clinicians suggested that a minimum of two years of continued implementation would be required to fully assess its long term impact on patient outcomes and healthcare utilisation. Proposed strategies to enhance sustainability included integrating CRM reviews within routine long term condition clinics to reduce duplication, offering shorter or less frequent follow up appointments to maintain momentum, and systematically tracking outcomes.

## Discussion

The study represents a real-world evaluation of a personalised, prevention-focused CRM pathway embedded within routine NHS primary care and demonstrates that a personalised, multidisciplinary primary care pathway can deliver measurable clinical benefits while also enhancing patient experience and staff development. Importantly the Harrow CRM hub highlights the pivotal role of primary care in early CRM identification and management, in the “real world”, demonstrating primary care to be the optimal setting for integrated chronic disease prevention. Across a diverse population of more than 2,600 patients, we observed significant reductions in blood pressure, HbA1c, and weight. Patients described the programme as distinct from routine care, valuing extended consultations, personalised explanations of test results, and culturally relevant advice. Clinicians reported increased confidence in health coaching and greater professional fulfilment. In parallel, the development of a bespoke educational framework equipped the workforce with the knowledge and skills required for holistic CRM care.

The clinical improvements observed are of clear significance. A mean systolic blood pressure reduction of nearly 14 mmHg aligns with international trial evidence, where reductions of just 5 mmHg are associated with ~ 10% fewer cardiovascular events. Similarly, a mean reduction of 9 mmol/mol in HbA1c is likely to be associated with significant reduction in long-term micro- and macro-vascular complications. Weight loss was modest at the population level, but even a 5% reduction is associated with lower risks of type 2 diabetes and cardiovascular disease – this needs to be recognised as occurring at the 3–6 month review post first appointment. Taken together, these findings suggest that embedding personalised CRM care in primary care can deliver health benefits at both the individual and population level. By showing that a local pathway can achieve improvements across all three domains, a personalised healthcare approach is clearly needed to be more widely adapted to reduce long term risk of CKD amongst high risk individuals.

In contrast to routine primary care, where patients are often reviewed through annual disease specific clinics (for example annual diabetes or hypertension reviews) with shorter consultation times, the CRM model brought multiple risk factors together into a single extended consultation. This reduced fragmentation of care, minimised duplication of appointments, and enabled a more holistic, patient centred discussion of risk, prevention, and management. A distinctive feature of the CRM model was its emphasis on patient engagement – the 30 min first appointment – engaging, listening, explaining and understanding. The primary care clinical model involved addressing a number of different clinical areas, e.g. BP, Cholesterol, medicines optimisation – rather than bringing the patient back for a number of different appointments which tends to be the current care delivery model in the UK. Patients described the extended consultations as transformative, allowing time to build trust, understand risk, and co-create actionable care plans. The use of visual explanations and culturally tailored advice enhanced engagement. This aligns with international evidence that culturally appropriate education is crucial for reducing disparities in diabetes and CKD outcomes. Importantly, some patients reported sharing knowledge with family members, suggesting a wider “ripple effect” on household health behaviours, with the potential for intergenerational benefits given the strong association between parental and childhood lifestyle patterns. These findings align closely with the principles of personalised healthcare, which emphasise shared decision-making, co-production of care plans, and support for self-management. By giving patients the time and tools to understand their individual risk profiles, the CRM pathway transformed abstract risk factors into meaningful and actionable steps. In turn, this built motivation, accountability, and confidence Importantly, these personalised elements were not add-ons but were built into the design of the pathway, demonstrating that personalisation can be systematically delivered within primary care. Moreover, the principles and communication skills instilled in CRM-trained clinicians have the ability to extend beyond the programme itself, influencing the quality of interactions across a wide range of consultations including mental health, women’s health, musculoskeletal, post-cancer, and frailty reviews reinforcing a more holistic, person-centred approach to care in primary care. In this sense, the Harrow CRM Hub offers a practical example of how the policy aspiration of personalised healthcare can be operationalised in a way that is both acceptable to patients and feasible for clinicians.

Clinicians consistently described the CRM clinics as more rewarding than routine reviews. The longer consultations, holistic focus, and structured templates allowed them to practise medicine in a way that felt both preventative and meaningful. The training programme, with its emphasis on shadowing and role-play, was especially valued for building confidence in lifestyle medicine and health coaching. These findings are consistent with wider literature showing that personalised care approaches can improve workforce morale and reduce burnout. The creation of a tiered educational framework represents a significant innovation, providing a scalable framework to support CRM care across primary care networks.

The Harrow cohort reflects the ethnic diversity of North West London, with substantial representation of South Asian and Black populations. Subgroup analyses revealed that South Asian patients achieved greater improvements in HbA1c, whereas White patients were more likely to achieve weight loss. Such differences highlight the need for tailored approaches to behaviour change and risk management, consistent with Core20PLUS5 and other NHS strategies to address health inequalities. Embedding personalised, culturally relevant care within primary care teams may help mitigate longstanding disparities in CKD detection and progression. By engaging a high-risk, ethnically diverse population and tailoring interventions to cultural and behavioural context, the CRM model directly contributes to reducing health inequalities in CKD and cardiometabolic outcomes.

This was a real-world evaluation conducted in routine primary care, recognising that not all patients demonstrated improvement across clinical parameters. Approximately 26% of patients showed some deterioration at early follow-up; however, these individuals were proactively reviewed within 3–6 months rather than waiting a full year for reassessment. This enabled timely optimisation of therapy, reinforcement of lifestyle strategies, and review of adherence and engagement. Importantly, implementation of the CRM pathway also led to wider improvements in clinical coding, particularly for hypertension, early liver disease, and early CKD. These enhancements have improved the accuracy of local prevalence estimates and facilitated earlier identification of risk in younger populations.

Strengths of this evaluation include the large and ethnically diverse cohort, the integration of quantitative and qualitative data, and the inclusion of workforce development as a core outcome. Unlike many disease-specific programmes, this study addressed the overlapping burden of obesity, diabetes, cardiovascular disease, and CKD as a single condition cluster, with the long-term aim of enabling unified care and reducing NHS resource use compared with traditional, siloed models.

Limitations include variable data completeness particularly for waist circumference limited follow-up duration restricting insight into long-term CKD progression and cardiovascular events, and uncertainty regarding the persistence of observed improvements in metabolic and vascular parameters. Qualitative findings primarily reflect the views of engaged participants and may not capture the perspectives of those who declined to participate. Variability in delivery between sites may also have influenced both outcomes and generalisability. Further analysis of patients demonstrating deterioration across clinical parameters was beyond the scope of this evaluation but represents an important area for future work to better understand variability in response.

Sustainability was a recurring theme in both patient and staff feedback. While most participants valued the model, challenges included missed appointments, incomplete pre-consultation questionnaires, and variation in practice-level engagement. Staff raised concerns regarding time pressures and long-term funding, consistent with previous evaluations of enhanced primary care programmes. Nevertheless, the potential system-wide benefits are considerable: improved risk-factor control may reduce demand for dialysis and cardiovascular admissions, while the integrated approach could streamline care, reduce duplication, and enhance continuity. Embedding CRM clinics within routine quality frameworks, supported by workforce training and digital tools, provides a practical pathway to slow the expected rise in CKD and CVD. This new model of delivery supports integrated care in the neighbourhood model and if anything underpins a new way to structure and support the anticipated Modern Service Framework for Cardiovascular (anticipated in early 2026), a key component of the 10 year plan of reducing cardiovascular deaths and morbidity with a focus on prevention. This model focuses on the further reduction of microvascular disease – by motivating the patient. Do you know your heart age?

The Harrow CRM Hub recognises that the details in this paper at the time of writing reflects our findings at month 9 into a 24 month programme but offers robust early evidence that personalised, multidisciplinary care can be embedded successfully within UK primary care, at scale and deliver clinically significant improvements in metabolic and cardiovascular risk factors. Its success rests on three key pillars: structured identification and monitoring, co-produced personalised care plans, and workforce empowerment through education. Future work should examine long-term renal and cardiovascular outcomes, cost-effectiveness, and implementation strategies for broader adoption. By aspiring to achieve national priorities while addressing local population needs, the CRM Hub bridges the gap between policy aspiration and clinical reality. If sustained and scaled, this model has the potential to slow CKD progression, reduce cardiovascular events, and most importantly enhance patient wellbeing across diverse communities and generations.

## Conclusion

A personalised, multidisciplinary CRM pathway embedded within primary care was associated with clinically and statistically significant improvements in blood pressure, glycaemic control, and weight across a large, ethnically diverse cohort. Patients reported greater activation and confidence, while clinicians gained new skills and fulfilment supported by a bespoke educational framework and integrated specialist input. Early system benefits included improved CRM recognition and coding accuracy, facilitating structured staging from Stage 0 to Stage 4 through established QOF registers and enhancing visibility of at-risk patients across Harrow.

Wider adoption of this model has the potential to strengthen all levels of patient care from individual motivation and self-management to population level insight and therefore improved system planning while embedding prevention into everyday clinical practice. If wider adoption is supported through sustained investment, workforce training, and improved utilisation of standardised digital tools, the CRM pathway could form a scalable and cost effective framework to slow CKD progression, reduce cardiovascular events, and meaningfully narrow inequalities across high risk and underserved populations.

## Supplementary Information

Below is the link to the electronic supplementary material.

Supplementary Material 1

## Data Availability

The datasets generated and/or analysed during this service-provision study are not publicly available, to ensure patient confidentiality. Summarised or aggregated data may be considered by the corresponding author on reasonable individual request, subject to appropriate governance processes.

## Abbreviations

ADA: sAmerican Diabetes Association
BMI: Body Mass Index
BP: Blood Pressure
CKD: Chronic Kidney Disease
CRM: Cardiorenal Metabolic
CVD: Cardiovascular Disease
DM: Diabetes Mellitus
EMIS: Egton Medical Information Systems
ESC: European Society of Cardiology
HbA1c: Haemoglobin A1c
ICB: Integrated Care Board
KDIGO: Kidney Disease: Improving Global Outcomes
KFRE: Kidney Failure Risk Equation
MECC: Making Every Contact Count
NHS: National Health Service
NWL: North West London
PCN: Primary Care Network
QOF: Quality and Outcomes Framework
QRISK2/QRISK3: Cardiovascular Risk Prediction Tools
RAAS: Renin–Angiotensin–Aldosterone System
SGLT2i: Sodium–Glucose Cotransporter-2 Inhibitor
SNOMED: Systematied Nomenclature of Medicine

## References

[1] Marassi M, Fadini GP. The cardio-renal-metabolic connection: a review of the evidence. Cardiovasc Diabetol. 2023;22(1):195. 10.1186/s12933-023-01937-x. PMID: 37525273; PMCID: PMC10391899. 37525273 10.1186/s12933-023-01937-x PMC10391899

[2] Xie K, Cao H, Ling S, Zhong J, Chen H, Chen P, Huang R. Global, regional, and national burden of chronic kidney disease, 1990–2021: a systematic analysis for the global burden of disease study 2021. Front Endocrinol (Lausanne). 2025;16:1526482. PMID: 40110544; PMCID: PMC11919670. 40110544 10.3389/fendo.2025.1526482 PMC11919670

[3] Public Health England. Chronic kidney disease prevalence model – 2021 update. London: PHE. 2021.Available from: https://assets.publishing.service.gov.uk/media/5a82c379e5274a2e8ab593af/ChronickidneydiseaseCKDprevalencemodelbriefing.pdf. Accessed 1/10/2025.

[4] Zhang H, et al. Global burden of metabolic diseases, 1990–2021. Metabolism. 2024;160:155999. 10.1016/j.metabol.2024.155999. Epub 2024 Aug 14. PMID: 39151887. 39151887 10.1016/j.metabol.2024.155999

[5] Berrington de Gonzalez A, et al. Body-mass index and mortality among 1.46 million white adults. N Engl J Med. 2010;363(23):2211-9. 10.1056/NEJMoa1000367. Erratum in: N Engl J Med. 2011;365(9):869. PMID: 21121834; PMCID: PMC3066051. 10.1056/NEJMoa1000367 PMC3066051 21121834

[6] Rao Kondapally Seshasai S, et al. Emerging Risk Factors Collaboration. Diabetes mellitus, fasting glucose, and risk of cause-specific death. N Engl J Med. 2011;364(9):829–841. 10.1056/NEJMoa1008862. Erratum in: N Engl J Med. 2011;364(13):1281. PMID: 21366474; PMCID: PMC4109980. 10.1056/NEJMoa1008862 PMC4109980 21366474

[7] Gansevoort RT, et al. Chronic kidney disease and cardiovascular risk: epidemiology, mechanisms, and prevention. Lancet. 2013;382(9889):339–52. 10.1016/S0140-67361360595-4. Epub 2013 May 31. PMID: 23727170. 10.1016/S0140-6736(13)60595-4 23727170

[8] Public Health England. Health matters: combating high blood pressure. London: Public Health England. 2017. Available from: https://www.gov.uk/government/publications/health-matters-combating-high-blood-pressure.

[9] NHS England (London Region). Diabetes: understanding the challenge in London. London: NHS England. 2015. Available from: https://www.england.nhs.uk/london/wp-content/uploads/sites/8/2019/07/dia-understanding-ldn-challenges-022015.pdf.

[10] Public Health England. Chronic kidney disease prevalence and cost model – 2021 update. London: Public Health England. 2021. Available from: https://digital.nhs.uk/data-and-information/publications/statistical/health-survey-for-england/2022-part-2/kidney-disease#undiagnosed-ckd.

[11] KDIGO Diabetes Work Group. KDIGO 2022 Clinical Practice Guideline for Diabetes Management in Chronic Kidney Disease. Kidney Int. 2022;102(5S):S1–127. 36272764 10.1016/j.kint.2022.06.008

[12] American Diabetes Association Professional Practice Committee. Diagnosis and classification of diabetes: Standards of Care in Diabetes—2025. Diabetes Care. 2025 Jan 1;48(Suppl 1):S27-S49 10.2337/dc25-S002. PMID: 39651986; PMCID: PMC11635041. 10.2337/dc25-S002 PMC11635041 39651986

[13] Marx N, Federici M, Schütt K, Müller-Wieland D, Ajjan RA, Antunes MJ, Christodorescu RM, Crawford C, Di Angelantonio E, Eliasson B, Espinola-Klein C, Fauchier L, Halle M, Herrington WG, Kautzky-Willer A, Lambrinou E, Lesiak M, Lettino M, McGuire DK, Mullens W, Rocca B, Sattar N, ESC Scientific document group. 2023 ESC guidelines for the management of cardiovascular disease in patients with diabetes. Eur Heart J. 2023;44(39):4043–4140. 10.1093/eurheartj/ehad192. Erratum in: Eur Heart J. 2023 Dec 21;44(48):5060. 10.1093/eurheartj/ehad774. Erratum in: Eur Heart J. 2024 Feb 16;45(7):518. https://doi.org/10.1093/eurheartj/ehad857. PMID: 37622663.

[14] Iacoviello M, Gori M, Grandaliano G, Minutolo R, Pitocco D, Trevisan R. A holistic approach to managing cardio-kidney metabolic syndrome: insights and recommendations from the Italian perspective. Front Cardiovasc Med. 2025;12:1583702. 10.3389/fcvm.2025.1583702. PMID: 40264513; PMCID: PMC12011791. 40264513 10.3389/fcvm.2025.1583702 PMC12011791

[15] National Institute for Health and Care Excellence (NICE). Chronic kidney disease: assessment and management (NG203). London: NICE. 2021. Available from: https://www.nice.org.uk/guidance/ng203. 34672500

[16] National Institute for Health and Care Excellence (NICE). Type 2 diabetes in adults: management (NG28). London: NICE; updated 2022. Available from: https://www.nice.org.uk/guidance/ng28. 32023018

[17] National Institute for Health and Care Excellence (NICE). Cardiovascular disease: risk assessment and reduction, including lipid modification (CG181)*.* London: NICE; updated 2023. Available from: https://www.nice.org.uk/guidance/cg181. 32200592

[18] The King’s Fund. Improving clinical co-ordination of care for people with multiple long-term conditions: the art of the possible. London: The King’s Fund. 2022. Available from: https://www.kingsfund.org.uk/publications/improving-clinical-co-ordination-care-multiple-long-term-conditions.

[19] Ortiz A, Arreola Guerra JM, Chan JCN, Jha V, Kramer H, Nicholas SB, Pavkov ME, Wanner C, Wong LP, Cheung M, King JM, Grams ME, Jadoul M, Tuttle KR, Conference Participants. Preventing chronic kidney disease and maintaining kidney health: conclusions from a kidney disease: improving global outcomes (KDIGO) controversies conference. Kidney Int. 2025;108(4):555–571. 10.1016/j.kint.2025.04.005. Epub 2025 Jun 19. PMID: 40536455. 10.1016/j.kint.2025.04.005 PMC12646115 40536455

[20] Gpcontract.co.uk. QOF Database: NHS England [Internet]. 2025 [accessed 12/11/2025]. Available from: https://www.gpcontract.co.uk/.

[21] Coulter A, Entwistle VA, Eccles A, Ryan S, Shepperd S, Perera R. Personalised care planning for adults with chronic or long-term health conditions. Cochrane Database Syst Rev. 2015;2015(3):CD010523. 10.1002/14651858.CD010523.pub2. PMID: 25733495; PMCID: PMC6486144. 25733495 10.1002/14651858.CD010523.pub2 PMC6486144

[22] NHS England. Personalised care: health coaching and peer support framework. London: NHS England. 2020. Available from: https://www.england.nhs.uk/personalisedcare/health-coaching/.

[23] NHS England. The NHS long term plan. London: NHS England. 2019. Available from: https://www.longtermplan.nhs.uk/.

[24] England NHS. Core20PLUS5: an approach to reducing healthcare inequalities. London: NHS England; 2022. Available from: https://www.england.nhs.uk/about/equality/equality-hub/core20plus5/.

