# Supplementary material for: A real-world preventive primary care model for cardiorenal metabolic disease: clinical impact of a personalised care approach in Harrow, North West London
Source: BMC Nephrol. 2026 May 29;27:450. doi: 10.1186/s12882-026-05040-7 (PMC13430914; doi:10.1186/s12882-026-05040-7)
Supplement: Supplementary file 1 — Supplementary Material 1 [file 12882_2026_5040_MOESM1_ESM.docx]

**Harrow CRM clinics
Patient engagement: Interviews**

**Audience:** patients who have recently attended a CRM clinic as part of their care

**Participants:** a mix of patients across the five participating PCN – as much as possible, we will aim to make sure we are speaking to a diverse range of participants in terms of age, gender, ethnicity, PCN.

**Platform:** Microsoft Teams

**Learning/evaluation aims:**

- To better understand the patient experience of the CRM clinics – what is working well and if there are areas for improvement.
- Ascertain if patients feel more informed, confident and motivated around managing their health and wellbeing.
- Enable the project team to identify opportunities to adapt the support offered if needed.

| **Introduction** | |
| --- | --- |
| **Introduce yourself and thank the participant for agreeing to be involved.**  **Tone and nature of the discussion**   - Clarify the length of interview – up to 1 hour. - Explain that there are no right or wrong answers. We are interested in hearing about their views and experiences. - We will be having similar chats with other patients too, and the calls will help us to identify opportunities to improve the service offered.     **Confidentiality**   - For the outputs of this activity, all comments will be **anonymized and your feedback will be combined with that of other patients we are speaking to. Whilst we may quote you in our final report, any quotes won’t be identifiable.** - **Confidentiality may be broken** if we feel there is immediate danger to yourself or another person or a safeguarding concern is raised. In this instance we would seek your consent to share. - **Ask for consent to record and transcribe the interview** so that you don’t have to make detailed notes. Recordings will be stored in secure folders to which only the interview team have access. Where consent is not provided, interviewer to seek consent to take detailed notes. - **Ask if they have any questions before you begin.** | |
| **#** | **Question** |
| **About them** | |
| **1** | **How long ago was it that you had an appointment at your practice about your overall health and well-being? We are referring to a new type of appointment that will have been a bit more in-depth and included a review of blood pressure, cholesterol, kidney function, and metabolism.**   - Ensure patient is aware of which appointment we mean. Patient will likely not be familiar with “CRM clinic” language. If need to describe appointment further, can add: this will have included discussion of diet, exercise, and lifestyle, and creation of a personalised plan to support your long-term health. |
| **Communication ahead of the appointment** | |
| **2.** | **How did you feel about being proactively contacted by your practice to come in for a health and well-being appointment?**   - Probe for whether messaging was clear, whether unhappy or happy to be contacted about this |
| **3.** | **How was the purpose of the appointment described to you?**   - Did you understand why you had been contacted? |
| ***4.** | **What, if anything, could have improved the communication you received ahead of the appointment?**   - Probe about any issues completing questionnaires or other asks/prerequisites before the appointment |
| **Condition management pre-appointment** | |
| **5.** | **Before the appointment, how confident did you feel managing your health and well-being?**   - If confident – what helped you to feel confident? - If not confident – what stopped you from feeling confident? |
| **6.** | **Before the appointment, how motivated did you feel to manage your health and well-being?**   - As above probe around what helped / got in the way of them feeling motivated. |
| ***7.** | **Before this recent appointment, had you ever had a conversation with a health professional about how you can better manage your health and wellbeing?**   - *If yes*, who was this with? What did the conversation involve? How helpful did you find it? |
| **Experience of the appointment** | |
| **8.** | **Who was the appointment with?** *(GP, pharmacist etc., not the individual)* |
| **9.** | **How comfortable did you feel talking to them about your health goals and any challenges you face in managing your health?**   - Is there anything that could have helped you to feel more comfortable? |
| ***10.** | **How helpful did you find the discussion?**   - What was most helpful about it? - How could it have been improved? |
| **11.** | **What were the key messages you took away from the appointment?**   - Is there anything that has stuck with you? |
| ***12.** | **Did you come away from the appointment with a clear understanding of your care plan and how it supports your health, well-being and daily independence?**   - How, if at all, has your understanding of your health condition(s) changed? |
| **Behaviour changes since the appointment** | |
| **13.** | **Following the appointment, how confident do you feel making some of the changes discussed?** |
| **14.** | **Compared to before the appointment how motivated do you feel to manage your health and well-being?**  **What changes, if any, have you made to how you manage your health since the appointment?**   - If they have made changes – what motivated you to make that / these change(s)? - If they haven’t made any changes – do you think that you will make any changes? Why/why not? |
| ***15.** | **GP Practices involved in delivering these health and wellbeing clinics are considering other ways they can continue to support patients with their health and wellbeing.**  **If you had the choice of an in-person or online instructor led education session or any anytime access pre-recorded podcast which would you prefer and why?**  In-person sessions would be about 1 hour each, with six sessions in total.   - If they have a preference for an instructor led education session probe around any preference between in-person vs online. |
| ***16.** | **Overall, how was your experience?**   - Would you be likely to attend a similar appointment again? - Is there anything else about your experience, not covered already, that you would like to share before we bring the call to a close? |
| **Thank you and close** | |
|  | Thank the participant for getting involved and for sharing their experiences.  Remind them that ICHP are offering a £25 in recognition of their time and contributions. This can either be in the form of a voucher or a bank transfer. They will receive an email about how to claim in the next few days. |

**Adaptation for on-site rapid interviews**

| **Introduction** |
| --- |
| **Introduce yourself, explain what we are doing, gain consent:**   - We are helping your healthcare provider to improve and to ask for your honest feedback about your experience with your appointment. - This feedback would be confidential and put into a summary with other patients’ feedback so that it is not attributable to you. - Confidentiality may be broken if we feel there is immediate danger to yourself or another person or a safeguarding concern is raised. In this instance we would seek your consent to share. - Would you be willing to speak with us for a few minutes for us to ask you a few questions about your thoughts?   - If not, might you be interested in speaking to us at another time, online using microsoft teams?   **Tone and nature of the discussion**   - Clarify the length of interview – 30 minutes if patient is free, otherwise can be as short as 5 minutes. - Explain that there are no right or wrong answers. We are interested in hearing about their views and experiences. - We will be having similar chats with other patients too, and the calls will help us to identify opportunities to improve the service offered. - Ask for consent to take audio recording just for notes purposes. Recordings will be stored in secure folders to which only the interview team have access. Where consent is not provided, interviewer to seek consent to take detailed notes.     **Ask if they have any questions before you begin.**  **Question list: questions in bold are priority:**   1. X 2. X 3. X |
|  |

**Harrow CRM clinics
Staff engagement: Interviews**

**OPERATIONAL STAFF**

**Participants:** operational staff e.g. practice managers, care coordinators

**Dissemination channel:** to be shared via Steven with practice clinical leads

**Platform:** Microsoft Teams

**Learning/evaluation aims:**

- Understand impact on operational staff of participation in CRM clinics project in terms of skills and knowledge
- Understand any operational barriers and enablers to delivering CRM clinics as intended in the project
- Enable the project team to adapt and target further support where needed
- Start to build blueprint of what works in terms of successful PCN/practice implementation, towards potential further rollout

| **Thank the participant for agreeing to be involved.**  **Tone and nature of the discussion**   - Clarify the length of interview – up to 45 minutes (at most). - Explain that there are no right or wrong answers. We are interested in hearing about their views and experiences of delivering CRM clinics. - We will be having similar conversations with other operational staff involved in the delivery of CRM clinics as well as with frontline staff involved in supporting the roll-out of this new way of working.     **Confidentiality**   - At the reporting stage, aggregated findings will be collated and presented in a short report. No individuals will be identified in the reporting and any quotes used will not be linked to any individual. - Everything they say is non-attributable. Check if they consent to be quoted – and if they would be happy to put their job role alongside any quotes. - Ask for consent to record and transcribe the interview so that you don’t have to make detailed notes. Recordings will be stored in secure folders to which only the interview team have access. Where consent is not provided, interviewer to seek consent to take detailed notes. - Ask if they have any questions before you begin. | |
| --- | --- |
| **#** | **Question** |
| **About them** | |
| **1** | What is your current role and involvement in implementing the CRM clinics? |
|  |  |
| **2** | Prior to the CRM clinics, have you had any previous experience of implementing a new care pathway? |
|  |  |
| **Experience of implementation** | |
| **3** | Has your practice started to roll-out the CRM clinics? |
|  |  |
| ***4** | [If yes] Can you share what this has this looked like so far?   - Probe on:   - Number of clinics held, how they are contacting patients, format of consultations etc.   - Experience of running centrally created searches to identify target cohort   - Methods of contacting patients & effectiveness of different types of messaging   - Method for collecting initial samples ahead of the clinic (bloods and urine) & effectiveness of approach |
|  |  |
| **5** | How have you found operationalising the CRM clinics in your practice/PCN?   - Probe on: has this been easy? Difficult? |
|  |  |
| **6** | What has helped enable you to do this?   - Probe: templates; guidance; training; communication - Any existing processes/norms at their practice that they have been able to loop this onto so that it doesn’t feel like added work |
|  |  |
| ***7** | What, if anything, has got in the way of being able to deliver as intended?   - Probe around what could help overcome these challenges - Probe around any barriers to engaging patients in particular, and what contact methods etc they are trying |
|  |  |
| **8** | What kind of guidance and support have you received in order to help set up these new clinics? How have you found that guidance and support?   - E.g. any trainings or communication on what the clinics are, how they are to be set up. - E.g. the “Harrow CRM Practice Guide” (7-page Word document with steps to follow around which patients to invite, how to set up clinics, how to do the reporting expected, etc), the “CRM simple guide” (3-page word document), the “CRM invite script (3-page Word document with verbal script to use when inviting patients to clinic). - Probe as to whether the info in the guides/sessions has been sufficient to set up clinics. - Probe around what elements of the support have been most helpful and what additional support, if any, would be helpful in operationalising this model of care. - Look for any differences between support/information received from the central team (e.g. guidance for all practices to follow), versus any **local support** e.g. from their practice manager, or immediate local colleagues (including their practice’s GPs/partners if they are not a GP). |
|  |  |
| **9** | Do you feel you have increased your knowledge of co-morbidity prevention and management?   - How confident do you feel responding to questions from patients around why they are being invited? What could increase your confidence?   How else has the support offered impacted on your skills and knowledge? |
|  |  |
| ***10** | If you were in charge of rolling this out to further practices and PCNs in another area, (a) would you support that idea?, and (b) if so what would be your top recommendations?   - This could be things that have gone well in their local rollout, or things they would do differently. |
|  |  |
| ***11** | Do you think these clinics are likely to continue beyond the project duration? Why or why not?   - May need to remind them that this is anonymised and they can be honest   We are asking as we know it is difficult for initiatives to be sustained, and we want to get an honest view of what might help this to be sustained |
|  |  |
| **Benefits** | |
| **12** | Have there been any benefits for you in your role, of being involved in the CRM clinics?   - If not mentioned, prompt around:   - Whether it has added to or reduced their workload.   - Impact on personal satisfaction in their role.   - If this approach has helped them acheive other targets the practice needs to meet e.g. QOF and enhanced services. |
|  |  |
| **13** | What benefits, if any, have you noticed for patients and clinical staff delivering the clinics? |
|  |  |
| **Thank you and close** | |
| - Give the interviewee an opportunity to share any final comments or reflections. - Thank the interviewee for their time and contributions. - Outline the next steps: the feedback you have shared today will contribute to an interim evaluation of the CRM clinic roll-out, helping to identify opportunities to enhance the support offered and facilitate successful implementation. | |

**Harrow CRM clinics
Staff engagement: Interviews**

**FRONTLINE STAFF**

**Audience:** delivery staff across participating PCNS – approx. 66 staff (1-2 staff at each of 33 practices)

**Participants:** a mix of staff roles and coverage from across participating PCNs. Survey responses will hopefully enable us to ensure we are speaking to people with a mix of experiences.

**Platform:** Microsoft Teams

**Learning/evaluation aims:**

- Ascertain staff confidence to deliver CRM clinics as intended in the model & identify any barriers
- Understand how useful staff perceive the Education Framework to be
- Understand what further learning/support staff may require in order to deliver CRM clinics
- Enable the project team to adapt and target further support where needed

| **Introduction** | |
| --- | --- |
| **Thank the participant for agreeing to be involved.**  **Tone and nature of the discussion**   - Clarify the length of interview – up to 45 minutes (at most). - Explain that there are no right or wrong answers. We are interested in hearing about their views and experiences of delivering CRM clinics. - We will be having similar conversations with other frontline staff involved in the delivery of CRM clinics as well as with operational staff involved in supporting the roll-out of this new way of working.     **Confidentiality**   - At the reporting stage, aggregated findings will be collated and presented in a short report. No individuals will be identified in the reporting and any quotes used will not be linked to any individual. - Everything they say is non-attributable. Check if they consent to be quoted – and if they would be happy to put their job role alongside any quotes. - Ask for consent to record and transcribe the interview so that you don’t have to make detailed notes. Recordings will be stored in secure folders to which only the interview team have access. Where consent is not provided, interviewer to seek consent to take detailed notes. - Ask if they have any questions before you begin. | |
| **#** | **Question** |
| **About them** | |
| **1** | What is your current role and involvement in delivering the CRM clinics?   - Approximately how many clinics have you delivered since this model was implemented? - Prior to the CRM clinics, have you had any previous experience of delivering a health coaching or personalised care approach? |
|  |  |
| **Training and learning** | |
| **2** | Which aspects of the CRM training and learning offer have been most helpful to you? Please provide specific examples.   - Probe on whether they have had opportunity to shadow a colleague |
|  |  |
| **3** | What improvements, if any, would you suggest for the current training and learning offer? |
|  |  |
| **4** | Have you faced any barriers that have prevented you from fully engaging with the training and learning opportunities? |
|  |  |
| **5** | Are there any additional training topics or support resources that would help you to feel more confident in delivering CRM clinics?   - Probe around ‘why’ for any suggestions given and what format they would like these covered in. |
|  |  |
| **Application of learning** | |
| **6.** | What have been the key things you have taken-away from the training and learning you have done so far? |
|  |  |
| **7** | Since the training have you had an opportunity to apply your learning to your CRM clinics?   - ***If yes,*** probe around what this has looked like e.g. How have you changed your approach? What have you done differently because of the training and why? - ***If no,*** what has stopped you from applying your learning? Is there anything you might want to consider doing differently because of the training / learning? |
|  |  |
| **8** | How confident have you felt applying the skills and knowledge learnt to your CRM clinics? |
|  |  |
| **Implementation of CRM clinics** | |
| **9** | How has your experience been delivering the CRM clinics so far? Have you been able to follow the guidance in the Standard Operating Procedure to implement the clinics in your practice?   - Probe on whether have invited both target cohorts of patients and any differences in how effective they feel the intervention has been for each - Probe on whether they feel they have been able to implement personalised care approach |
|  |  |
| **10** | What has helped enable you to do this? |
|  |  |
| **11** | What, if anything, has got in the way of being able to deliver as intended?   - If not covered already, probe around whether they have been able to cover all clinical areas in the consultation. - Probe around what could help overcome these challenges |
|  |  |
| **12** | If you were in charge of rolling this out to further practices and PCNs in another area, what would be your top recommendations?   - This could be things that have gone well in their local rollout, or things they would do differently. - Prompts:   - Which roles have been delivering the clinics   - Consultation time   - Cohort   - Techniques used in consultations; what’s worked best |
|  |  |
| **Benefits** | |
| **13** | What have been the key benefits of this approach for you as a healthcare professional? |
|  |  |
| **14** | What benefits, if any, have you noticed for your patients? |
|  |  |
| **Thank you and close** | |
| - Give the interviewee an opportunity to share any final comments or reflections. - Thank the interviewee for their time and contributions. - Outline the next steps: the feedback you have shared today will contribute to an interim evaluation of the CRM clinic roll-out, helping to identify opportunities to enhance the training and support offered and facilitate successful implementation. | |

**Harrow CRM clinics
Staff engagement: Survey**

**FRONTLINE STAFF**

**Audience:** delivery staff across participating PCNS – approx. 66 staff (1-2 practice leads at each of 33 practices)

**Dissemination channel:** to be shared with relevant staff via Steven

**Platform:** below draft questions to be set up as a survey in Microsoft Forms by ICHP team

**Learning/evaluation aims:**

- Ascertain staff confidence to deliver CRM clinics as intended in the model & identify any barriers
- Understand how useful staff perceive the Education Framework to be
- Understand what further learning/support staff may require in order to deliver CRM clinics
- Enable the project team to adapt and target further support where needed

**Option to participate in more depth:** The survey will seek participants’ consent to be contacted to share more detailed insights via 1-1 interviews and/or focus groups.

| **Staff Survey – Cardio Renal Metabolic (‘CRM’) Clinics** |
| --- |
| **Introduction** |
| **What is the purpose of this survey?**  Thank you in advance for sharing your thoughts in this survey. This survey is intended for Harrow staff delivering clinics to patients via the new cardio-renal-metabolic (‘CRM’) clinics. Your feedback will help the Harrow CRM programme team to:   - Learn what works and doesn’t work for you, to best enable you and future staff to deliver this type of care to patients - Understand your level of confidence in delivering cardio-renal-metabolic clinics - Understand what types of learning you find most useful, including whether you find the education framework useful - Adapt and target support to where it is needed   Please be as honest as possible, as the team is very keen to know where staff may need more support to deliver this new model of care and benefit patients.  **Who will see my survey responses?**  This survey is being carried out by Imperial College Health Partners (ICHP), the Health Innovation Network for North West London, who are helping to evaluate the CRM clinics on behalf the Harrow CRM programme team.  All responses will be treated in the strictest confidence and there is the option to complete the survey anonymously.  The survey findings will be analysed by Imperial College Health Partners and the results will be presented in a summary report in which no individual or their responses can be identified. PCN level data will be used so that we can direct support to the right places where it’s needed, but your responses will not be identifiable.  **The survey includes 17 questions and will take under 10 minutes to complete.**    If you have any questions about the survey, please contact Imperial College Health Partners: [communications.engagement@imperialcollegehealthpartners.com](mailto:communications.engagement@imperialcollegehealthpartners.com). |
|  |

| **SECTION 1: About them** | **Internal project team notes** |
| --- | --- |
| 1. PCN    - Health Alliance    - Sphere    - Harrow Collaborative    - Harrow East    - Healthsense 2. Which of these best describes your role? :  - GP - Healthcare Assistant - Health coach - Practice Nurse - Non-prescribing clinician - Clinical Pharmacist - Other specialist - Advanced Clinical Practitioner - Other (please specify)  1. Approximately how many cardio-renal-metabolic (CRM) clinics have you delivered as of today?    - 0    - 1-10    - 11-20    - 21-30    - 31-40    - Greater than 40 | - We envision this to still allow anonymity as each PCN would have ~10 leads per PCN, but allow us to learn if particular PCNs need more support. Could do at practice level but that could effectively remove anonymity so would need to change expectations on that. - Role – may be useful to be able to cut responses based on this and look for any patterns - Useful to associate with other responses |
| **Overall confidence** |  |
| 1. I feel I know what is expected of me in delivering CRM clinics to patients as outlined in the Standard Operating Procedure and Practice Guide.    - Strongly agree    - Agree    - Neither agree nor disagree    - Disagree    - Strongly disagree 2. I feel confident in delivering CRM clinics to patients.    - Strongly agree    - Agree    - Neither agree nor disagree    - Disagree    - Strongly disagree 3. What would help you to feel more confident? [free text] | - Helps us identify and address a key potential barrier - Helps us take action / know how to better support staff |
| **Training and learning** |  |
| 1. To what extent would you find the following styles of learning helpful in enabling you to deliver CRM care to patients? [matrix, very helpful to very unhelpful]    - Self-directed learning, i.e. online modules and materials that I can read and watch in my own time    - Shadowing another colleague in real world settings, i.e. observing a colleague delivering a clinic to a patient    - Live lectures, i.e. a speaker presents information using structured visuals and verbal explanation    - Role-playing practice, i.e. I try something out and get feedback from colleagues 2. Have you attended one of the CRM Health Coaching Training sessions?    - Yes    - No    - Unsure 3. I feel the education framework (guide of independent learning modules) will better enable me to deliver CRM clinics/care to patients.    - Strongly agree    - Agree    - Neither agree nor disagree    - Disagree    - Strongly disagree    - I don’t know what the education framework is / haven’t seen it 4. Is there anything in the way of you completing the ‘required’ independent learning modules in the education framework? Tick all that apply.    - No, nothing is in the way    - I don’t have enough time    - It’s not clear to me how to utilise the framework    - I can’t access the courses    - It isn’t the type of learning that I need    - I am confident in my skills already    - Other, please specify 5. Is there anything you feel is missing from the education framework? [free-text, optional] 6. What additional training or support, if any, would be most helpful to you? [free-text, optional] | - Informs our blueprint of how to effectively enable staff. - See if there is association between session attendance and confidence, if we hypothesise this might be a key differentiator - Measure overall perception of framework as useful - Understand the barriers to using framework |
| **Impact of learning and project involvement** |  |
| 1. To what extent do you agree with the statements below? Since the start of my involvement in the CRM clinics: [Matrix, Strongly agree to strongly disagree with an n/a option]:    - I feel confident applying health coaching skills during clinics    - I have increased my knowledge around co-morbidity prevention and management    - I have a gained a greater awareness of local services I can signpost/refer patients to    - I feel more able to access the expertise of wider team members within my practice    - I feel more able to access the expertise of wider team members outside of my practice (e.g. renal consultant) 2. What impact has your involvement in CRM clinics had on overall satisfaction in your role/work so far?    - It has greatly increased my satisfaction    - It has increased my satisfaction    - It has not made a difference    - It has decreased my satisfaction    - It has greatly decreased my satisfaction 3. To what extent do you agree with the statements below? [Matrix, Strongly agree to strongly disagree with an n/a option]:    - I feel motivated to deliver the CRM clinics.    - I feel I have been able to deliver the CRM clinics as intended. 4. Are there any barriers that have stopped you delivering the clinics as intended? Tick all that apply.    - No, nothing is in the way    - I don’t have enough time    - Lack of an appropriate space    - Too much to cover with the patient in the time    - I would benefit from more training/knowledge    - I would benefit from more operational support    - Other, please specify 5. [Optional, free text] What additional operational support would help you to have more seamless CRM clinics? | - Awareness of local services – intention here is to measure greater knowledge of community resources per evaluation framework - Access expertise of wider team members – intended to capture sense of integrated working per evaluation framework - Staff satisfaction measure in evaluation framework - Surface potential barriers - Surface potential barriers |
| **Have your say** |  |
| To help us to understand how staff are currently finding the CRM clinics, we are looking to hold follow-up conversations to build on the insights shared through this survey. As part of this we would love to hear your views!   **Taking part will involve a short 30 minute call with a member of the Imperial College Health Partners (ICHP) team**. This will be arranged around your availability to ensure a convenient date and time.   Your involvement will be confidential  - all feedback shared will be **non-attributable** and combined with wider findings to ensure you feel able to contribute honestly.  These conversations will play an important role in helping to identify opportunities to enhance the training and support offered to staff delivering CRM clinics.  I would like to share my views and consent to being contacted by ICHP about a 1-1 interview:   - Yes - No   If you answered yes, please either provide your email address here, or if you prefer for your survey responses to remain anonymous, email [communications.engagement@imperialcollegehealthpartners.com](mailto:communications.engagement@imperialcollegehealthpartners.com) with subject line “CRM feedback”.  [single-line free text] |  |
